# Candidate New Rotavirus Species in Sheltered Dogs, Hungary

**DOI:** 10.3201/eid2104.141370

**Published:** 2015-04

**Authors:** Eszter Mihalov-Kovács, Ákos Gellért, Szilvia Marton, Szilvia L. Farkas, Enikő Fehér, Miklós Oldal, Ferenc Jakab, Vito Martella, Krisztián Bányai

**Affiliations:** Hungarian Academy of Sciences–Centre for Agricultural Research, Budapest, Hungary (E. Mihalov-Kovács, S. Marton, S.L. Farkas, E. Fehér, K. Bányai);; Hungarian Academy of Sciences–Centre for Agricultural Research, Martonvásár, Hungary (Á. Gellért);; University of Pécs, Pécs, Hungary (M. Oldal, F. Jakab);; Università Aldo Moro di Bari, Valenzano, Italy (V. Martella)

**Keywords:** viral metagenomics, viruses, Hungary, semiconductor sequencing, rotavirus, astrovirus, parvovirus, coronavirus, picodicistrovirus, vesivirus, dogs

## Abstract

We identified unusual rotavirus strains in fecal specimens from sheltered dogs in Hungary by viral metagenomics. The novel rotavirus species displayed limited genome sequence homology to representatives of the 8 rotavirus species, A–H, and qualifies as a candidate new rotavirus species that we tentatively named *Rotavirus I*.

Rotaviruses (family *Reoviridae*, genus *Rotavirus*) are major causes of acute dehydrating gastroenteritis in birds and mammals (*1*). Rotaviruses have an 11-segmented dsRNA genome encoding 6 structural proteins (viral protein [VP] 1–4, VP6, and VP7) and at least 5 functional nonstructural proteins (NSPs; NSP1–NSP5) (Technical Appendix Table 1). Traditionally, rotaviruses have been classified into (sero)groups on the basis of major antigenic differences that predominantly reside in the VP6 and of the genomic RNA profile obtained by polyacrylamide gel electrophoresis and silver staining (*1*). Recently, a VP6 gene sequence–based classification scheme has been proposed to replace the conventional methods. An empirical 53% aa identity was demonstrated to reliably distinguish strains of various rotaviruses groups (*2*). Also, reclassification of the 8 rotavirus groups as distinct species within the *Rotavirus* genus, designated *Rotavirus A–H*, has been proposed.

Rotavirus A has been detected in a wide variety of mammals and birds. In mammals, both endemic and epidemic forms of rotavirus B, C, E, and H infections have been described, whereas rotavirus D, F, and G have been identified only in birds (*1*–*3*). Genetically diverse rotaviruses have been found in some viral metagenomics studies (*4*,*5*). Using the metagenomic approach and the VP6-based molecular classification scheme, we found evidence for a novel rotavirus species that we tentatively called *Rotavirus I*.

## The Study

During 2012, we collected fecal specimens from sheltered dogs in northern Hungary to detect enteric viruses. Of 63 samples obtained from 50 animals, 37 randomly selected samples (from 33 animals) were subjected to random primed reverse transcription PCR and semiconductor sequencing by using the Ion Torrent PGM platform (New England Biolabs, Ipswich, MA, USA) (Technical Appendix). Bioinformatics analysis consisted of the mapping of reads >40 bases against ≈1.7 million viral sequences downloaded from GenBank by applying moderately rigorous mapping parameters (length fraction 0.6; similarity fraction 0.8) within the CLC Genomics Workbench (http://www.clcbio.com/).

One sample (KE135/2012) obtained from a suckling dog in May 2012 was positive for several enteric viruses. When analyzing the initially obtained ≈60.5-K sequence reads, in addition to canine rotavirus A (141 reads), astrovirus (2,399 reads), and parvovirus (3,623 reads), we identified a single 53-nt sequence read that mapped to the VP1 gene of rotavirus B. Another sample, KE528/2012, collected during August 2012 from an adult dog with diarrhea, was positive for coronavirus (30 reads), vesivirus (17 reads), picodicistrovirus (3 reads), and astrovirus (1 read); in addition, 7 and 5 sequence reads, respectively, mapped to the VP1 and VP3 genes of rotavirus H and/or B.

Subsequently, we enriched genomic dsRNA of KE135/2012 by differential LiCl precipitation; however, the enriched dsRNA remained invisible by polyacrylamide gel electrophoresis and silver staining. Because of the apparent low titer of the novel rotavirus, we tried to obtain more sequence data by drastically increasing the output in parallel sequencing runs. De novo assembly of the resulting ≈1.59 million sequence reads readily identified homologs of the structural and some nonstructural genes, which were divergent from rotavirus A–H reference sequences (Table; Technical Appendix Table 1). Determination of the coding regions in most cases was successful by extension of the termini of consensus sequences using the Ion Torrent sequence reads. However, we found no evidence for NSP3 and NSP4 with this approach, probably because of the great sequence divergence of these genes across members of the genus (*6*,*7*). Because the genomic RNA of each rotavirus species is characterized by low GC (guanine:cytosine) content (29%–40%), we expected that contigs with low GC content and with no GenBank homologs might be good candidates for detecting the missing genes. Indeed, further assembly and subsequent analysis of selected sequence stretches helped to identify the NSP3 by similarity search through the blastx engine (http://blast.ncbi.nlm.nih.gov/Blast.cgi) after an 800-bp long fragment was obtained, and analysis of the structural features of the deduced protein sequence supported detection of the putative NSP4. The obtained consensus sequence was used as reference to map other viral metagenomics data generated from the sheltered dog population; however, except for the aforementioned sample, KE528/2012, we found no additional specimens by this method to contain homologous viruses. The 2 related unusual rotaviruses, KE135/2012 and KE528/2012, had conserved genome segment termini (5′ end, GGC/TA; 3′ end, AACCC) and shared high sequence identities in most genes (e.g., VP2: 88% nt, 95% aa; NSP4: 99% nt, 99% aa) and very low sequence similarity in the VP7 gene (53% nt, 38% aa) (GenBank accession nos. KM369887–KM369908; Technical Appendix).

**Table:** Sequencing depth for the putative rotavirus I strains obtained by massively parallel sequencing*

Gene	KE135/2012			KE528/2012	
	Mapped read count	Average coverage (X)		Mapped read count	Average coverage (X)
VP1	9632	478		1286	59
VP2	7762	455		860	46
VP3	6361	510		657	49
VP4	5887	436		716	47
VP6	4762	700		582	72
VP7	2841	594		258	45
NSP1	3677	450		561	62
NSP2	2980	529		401	64
NSP3	2528	523		176	32
NSP4	2272	586		229	51
NSP5	1098	387		249	72

*Total sequence reads to obtain genomic RNA sequence for KE135/2012 and KE 528/2012 were 1,591,803, and 144,747, respectively. The minimum overlap with the consensus sequences (i.e., the de novo assembled rotavirus I–specific consensus sequences) was 20 nt, the minimum identity was 80%. To improve the mapping results, the following gap penalties were applied for the dataset: mismatch cost = 2, insertion cost = 3, deletion cost = 3. After visual inspection of the sequence alignments and remapping onto the obtained gene sequence, a single consensus sequence was finalized for each genome segment.

The deduced VP6 amino acid sequences served as the basis to classify these 2 unusual rotavirus strains (*2*). The greatest amino acid sequence identity of the VP6 proteins was found when compared to the novel rotavirus H strains (<46%); lower sequence similarities were found in comparison to randomly selected representatives of other rotvirus species (e.g., rotavirus G and B, <37%; rotavirus A, C, D, and F, <18%).

**Figure 1:**
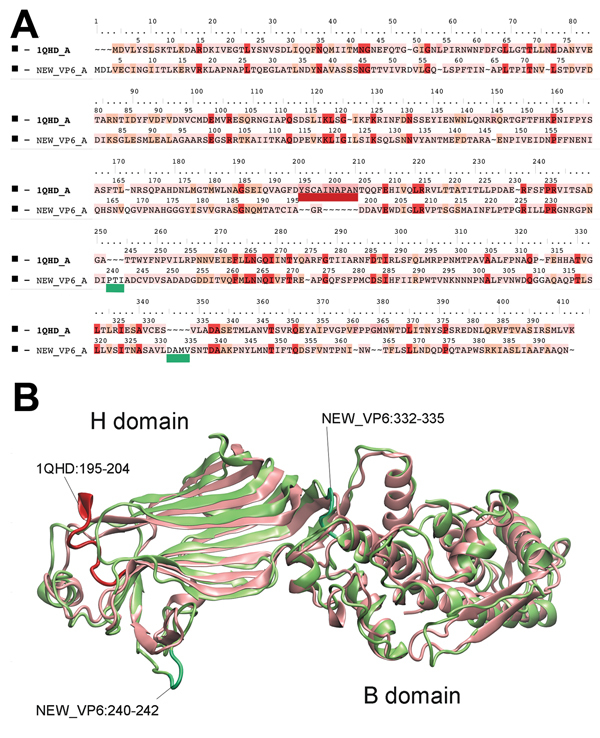
Structure comparison of rotavirus viral protein (VP) 6 proteins. A) Structure-based amino acid sequence alignment of the novel canine rotavirus VP6 protein and the template bovine rotavirus A VP6 protein. The background of the sequence alignments reflects the homology levels of the 2 VP6 sequences. Red, identical amino acid; orange, similar amino acid; pink, different amino acid). The main structural differences are indicated by dark red and menthol green on the sequence alignment and on the superimposed VP6 structures (B). Cartoon presentation of the homologous VP6 proteins: pink, rotavirus A; green, rotavirus I. Further information is available in the Technical Appendix.

**Figure 2:**
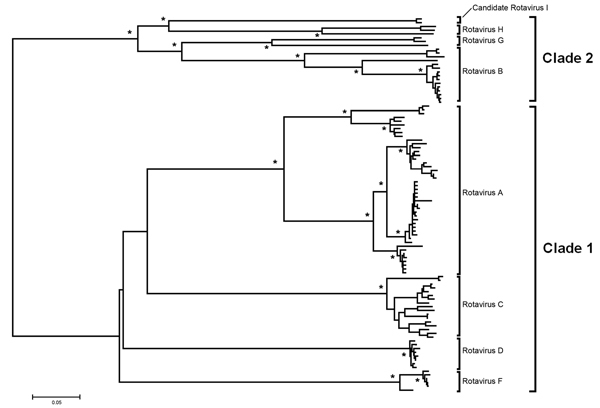
Protein sequence–based phylogenetic tree of the rotavirus viral protein 6 gene obtained by the neighbor-joining algorithm. Asterisks indicate >90% bootstrap values. The 2 canine rotavirus strains from Hungary that belong to the proposed novel *Rotavirus I* cluster with rotavirus H, G, and B within a major clade referred to as clade 2. Rotavirus A, C, D, and F strains belong to clade 1 (*6*). Scale bar indicates nucleotide substitutions per site.

To extend the analysis and assess whether the obtained VP6 gene might be functionally integral, we conducted molecular modeling of the amino acid sequence. In brief, amino acid sequence similarity values created a reliable protein model (*8*,*9*) showing similar protein folding of the VP6 monomer and comparable electrostatic charge pattern around the 3-fold axis of the VP6 homotrimer to that experimentally determined for rotavirus A (Figure 1). Subsequent phylogenetic analysis of the VP6 protein identified 2 major clades of rotaviruses (*6*). The novel rotavirus strains clustered with species H, G, and B within clade 2, whereas clade 1 included representative strains of species A, C, D, and F (Figure 2). This pattern of clustering was also evident when we analyzed the remaining genes. Collectively, sequence and phylogenetic analysis demonstrated moderate genetic relatedness of the unusual canine rotaviruses to representative strains of species A–H, suggesting that they belong to a novel species, tentatively called *Rotavirus I*. The prototype strains were named RVI/Dog-wt/HUN/KE135/2012/G1P1 and RVI/Dog-wt/HUN/KE528/2012/G2P1 according to recent guidelines (*10*) (online Technical Appendix).

Short rotavirus sequences detected recently in the fecal viral flora of cats and California sea lions (*4*,*5*) showed closer relatedness to our strains in the amplified VP6- and VP2-specific stretches, respectively, than to the corresponding genomic regions of reference rotavirus species (VP6, ≈70 aa, 67% vs. <55%; VP2, ≈160 aa, 78%–86% vs. <44%) (Technical Appendix). These published data (*4*,*5*) together with our results suggest that genetically related non–rotavirus A–H strains occur in various carnivore host species and geographic settings.

## Conclusions

We identified 2 representative strains of a novel rotavirus species, *Rotavirus I*. Many questions remain, including those related to the epidemiology, host range, and evolution of this species. One intriguing finding was the distantly related VP7 genes expressed on a fairly conserved genetic backbone. Typically, very low sequence identity values within the VP7 gene (e.g., rotavirus A, as low as 60% nt and 55% aa; rotavirus B, 54% nt, 46% aa; rotavirus H, 63% nt, 56% aa) can be seen when strains from different host species are compared (*11*–*13*). Whether the VP7 gene(s) of rotavirus I strains could have been acquired in the past from another rotavirus species by reassortment remains uncertain, given that reassortment among various rotavirus species is thought to occur only rarely (*7*,*14*). Further information is needed to better understand this genetic diversity within rotavirus I.

**Technical Appendix.** Comparison of the genome size and the coding potential of different rotavirus species; laboratory methods; percentile nucleotide and amino acid sequence-based identities between the novel canine rotavirus strain and reference rotavirus A–D and F–H strains; 5′ and 3′ termini confirmed by Sanger sequencing; additional insight into the structure of viral protein (VP) 6 and its homotrimer form; phylogenetic trees obtained for VP1–VP4, VP7, nonstructural protein (NSP) 1 to NSP5 proteins with representative strains of rotavirus A–H; and phylogenetic trees obtained for the partial sequences using unusual feline and otarine rotavirus gene sequences.

## References

[1] Estes MK, Kapikian AZ. Rotaviruses. In: Knipe DM, Howley PM, Griffin DE, Lamb RA, Martin MA, Roizman B, et al., editors. Fields virology. 5th ed. Philadelphia: Lippincott Williams & Wilkins; 2007. p. 1917–74.

[2] Matthijnssens J, Otto PH, Ciarlet M, Desselberger U, Van Ranst M, Johne R. VP6-sequence-based cutoff values as a criterion for rotavirus species demarcation. Arch Virol. 2012;157:1177–82. 10.1007/s00705-012-1273-3 22430951

[3] Marthaler D, Rossow K, Culhane M, Goyal S, Collins J, Matthijnssens J, Widespread rotavirus H in commercially raised pigs, United States. Emerg Infect Dis. 2014;20:1195–8 . 10.3201/eid2007.140034 24960190 PMC4073875

[4] Ng TF, Mesquita JR, Nascimento MS, Kondov NO, Wong W, Reuter G, Feline fecal virome reveals novel and prevalent enteric viruses. Vet Microbiol. 2014;171:102–11. 10.1016/j.vetmic.2014.04.005 24793097 PMC4080910

[5] Li L, Shan T, Wang C, Côté C, Kolman J, Onions D, The fecal viral flora of California sea lions. J Virol. 2011;85:9909–17. 10.1128/JVI.05026-11 21795334 PMC3196430

[6] Kindler E, Trojnar E, Heckel G, Otto PH, Johne R. Analysis of rotavirus species diversity and evolution including the newly determined full-length genome sequences of rotavirus F and G. Infect Genet Evol. 2013;14:58–67. 10.1016/j.meegid.2012.11.015 23237956

[7] Trojnar E, Otto P, Roth B, Reetz J, Johne R. The genome segments of a group D rotavirus possess group A–like conserved termini but encode group-specific proteins. J Virol. 2010;84:10254–65. 10.1128/JVI.00332-10 20631147 PMC2937790

[8] Roy A, Kucukural A, Zhang Y. I-TASSER: a unified platform for automated protein structure and function prediction. Nat Protoc. 2010;5:725–38. 10.1038/nprot.2010.5 20360767 PMC2849174

[9] Mathieu M, Petitpas I, Navaza J, Lepault J, Kohli E, Pothier P, Atomic structure of the major capsid protein of rotavirus: implications for the architecture of the virion. EMBO J. 2001;20:1485–97. 10.1093/emboj/20.7.1485 11285213 PMC145492

[10] Matthijnssens J, Ciarlet M, McDonald SM, Attoui H, Bányai K, Brister JR, Uniformity of rotavirus strain nomenclature proposed by the Rotavirus Classification Working Group (RCWG). Arch Virol. 2011;156:1397–413. 10.1007/s00705-011-1006-z 21597953 PMC3398998

[11] Matthijnssens J, Ciarlet M, Heiman E, Arijs I, Delbeke T, McDonald SM, Full genome-based classification of rotaviruses reveals a common origin between human Wa-Like and porcine rotavirus strains and human DS-1–like and bovine rotavirus strains. J Virol. 2008;82:3204–19 and. 10.1128/JVI.02257-07 18216098 PMC2268446

[12] Marthaler D, Rossow K, Gramer M, Collins J, Goyal S, Tsunemitsu H, Detection of substantial porcine group B rotavirus genetic diversity in the United States, resulting in a modified classification proposal for G genotypes. Virology. 2012;433:85–96. 10.1016/j.virol.2012.07.006 22877843 PMC7111968

[13] Wakuda M, Ide T, Sasaki J, Komoto S, Ishii J, Sanekata T, Porcine rotavirus closely related to novel group of human rotaviruses. Emerg Infect Dis. 2011;17:1491–3 . 21801631 10.3201/eid1708.101466 PMC3381553

[14] Esona MD, Mijatovic-Rustempasic S, Conrardy C, Tong S, Kuzmin IV, Agwanda B, Reassortant group A rotavirus from straw-colored fruit bat (*Eidolon helvum*). Emerg Infect Dis. 2010;16:1844–52. 21122212 10.3201/eid1612.101089 PMC3294550

