# Supplementary material for: Candidate New Rotavirus Species in Sheltered Dogs, Hungary
Source: Emerg Infect Dis. 2015 Apr;21(4):660–3. doi: 10.3201/eid2104.141370 (PMC4378476; doi:10.3201/eid2104.141370)
Supplement: Supplementary file 1 — Technical Appendix. Comparison of the genome size and the coding potential of different rotavirus species; laboratory methods; percentile nucleotide and amino acid sequence-based identities between the novel canine rotavirus strain and reference rotavirus A–D and F–H strains; 5′ and 3′ termini confirmed by Sanger sequencing; additional insight into the structure of viral protein (VP) 6 and its homotrimer form; phylogenetic trees obtained for VP1–VP4, VP7, nonstructural protein (NSP) 1 to NSP5 proteins with representative strains of rotavirus A–H; and phylogenetic trees obtained for the partial sequences using unusual feline and otarine rotavirus gene sequences. [file 14-1370-Techapp-s1.pdf]

Article DOI: http://dx.doi.org/10.3201/eid2104.141370

Candidate New Rotavirus Species in Sheltered Dogs, Hungary

Technical Appendix

Technical Appendix Table 1. Comparison of the genome size and the coding potential of different rotavirus species*

Genome segment	*Rotavirus A*, Wa		*Rotavirus A*, 02V0002G3		*Rotavirus B*, Bang373		*Rotavirus C*, Bristol		*Rotavirus D*, 05V0049	
	Length, nt	Protein (aa)	Length, nt	Protein (aa)	Length, nt	Protein (aa)	Length, nt	Protein (aa)	Length, nt	Protein (aa)
1	3302	VP1 (1088)	3305	VP1 (1089)	3511	VP1 (1160)	3309	VP1 (1090)	3274	VP1 (1079)
2	2717	VP2 (890)	2732	VP2 (895)	2847	VP2 (934)	2736	VP2 (884)	2801	VP2 (913)
3	2591	VP3 (835)	2583	VP3 (829)	2341	VP3 (763)	2283	VP3 (693)	2366	VP4 (777)
4	2359	VP4 (775)	2354	VP4 (770)	2306	VP4 (750)	2166	VP4 (744)	2104	VP3 (685)
5	1567	NSP1 (486)	2122	NSP1 (577)	1276	NSP1–1 (107)<br>NSP1–2 (321)<br>NSP1–3 (65)	1353	VP6 (395)	1872	NSP1 (574)
6	1356	VP6 (397)	1348	VP6 (397)	1269	VP6 (391)	1350	NSP3 (402)	1353	VP6 (398)
7	1074	NSP3 (310)	1089	NSP3 (304)	1179	NSP3 (347)	1270	VP6 (394)	1242	NSP3 (370)
8	1062	VP7 (326)	1066	VP7 (329)	1007	NSP2 (301)	1063	VP7 (332)	1026	NSP2 (310)
9	1059	NSP2 (317)	1042	NSP2 (315)	814	VP7 (249)	1037	NSP2 (312)	1025	VP7 (316)
10	750	NSP4 (175)	724	NSP4 (168)	751	NSP4 (219)	730	NSP5 (212)	765	NSP4 (127)<br>ORF2 (93)
11	664	NSP5 (197)<br>NSP6 (92)	699	NSP5 (208)	631	NSP5 (170)	615	NSP4 (150)	672	NSP5 (195)
Sum	18,501		19,064		17,932		17,912		18,500	

Genome segment	*Rotavirus F*, 03V0568		*Rotavirus G*, 03V0567		*Rotavirus H*, J19		*Rotavirus I*, KE135/2012		*Rotavirus I*, KE528/2012	
	Length, nt	Protein (aa)	Length, nt	Protein (aa)	Length, nt	Protein (aa)	Length, nt	Protein (aa)	Length, nt	Protein (aa)
1	3296	VP1 (1086)	3526	VP1 (1160)	3538	VP1 (1167)	3518	VP1 (1162)	3518	VP1 (1162)
2	2769	VP2 (904)	3014	VP2 (991)	2969	VP2 (973)	3002	VP2 (982)	3000	VP2 (982)
3	2246	VP4 (738)	2364	VP4 (772)	2512	VP4 (823)	2371	VP4 (777)	2370	VP4 (777)
4	2174	VP3 (694)	2352	VP3 (768)	2204	VP3 (719)	2161	VP3 (701)	2162	VP3 (701)
5	1791	NSP1 (547)	1295	NSP1–1 (106)<br>NSP1–2 (324)	1307	NSP1 (395)	1485	NSP1–1 (79)<br>NSP1–2 (390)	1484	NSP1–1 (79)<br>NSP1–2 (390)
6	1314	VP6 (396)	1267	VP6 (391)	1287	VP6 (396)	1278	VP6 (395)	1279	VP6 (395)
7	1309	NSP3 (370)	1052	NSP3 (300)	1004	NSP3 (297)	1018	NSP2 (301)	1016	NSP2 (301)
8	1068	NSP2 (318)	1012	NSP2 (282)	932	NSP2 (262)	954	NSP3 (273)	951	NSP3 (273)
9	990	VP7 (295)	825	VP7 (247)	820	VP7 (258)	858	VP7 (268)	869	VP7 (273)
10	706	NSP5 (218)	801	NSP4 (187)	739	NSP4 (213)	751	NSP4 (219)	750	NSP4 (219)
11	678	NSP4 (169 aa)	678	NSP5 (181)	649	NSP5 (176)	593	NSP5 (157)	589	NSP5 (157)
Sum	18,341		18,186		17,961		17,989		17,988	

*Rotavirus species and type strain is shown in the upper row. The coding regions were predicted using the ORF Finder program (http://www.ncbi.nlm.nih.gov/gorf/gorf.html).

Laboratory Methods

Semiconductor Sequencing

Ten percent fecal suspensions were prepared in phosphate buffered saline and then centrifuged at 5000 × g for 10 min. Viral RNA was extracted by using the Zymo DirectZol kit (Zymo Research, Orange, CA, USA) combined with the RiboZol RNA extraction ragent (Amresco, Solon, OH, USA), according to the protocol recommended by the manufacturer for biological liquids, although DNase treatment was omitted from the workflow.

The RNA sample was subsequently denatured at 97°C for 5 min in the presence of 10 µM random hexamer tailed by a common PCR primer sequence (*1*). Reverse transcription was performed with 1 U AMV reverse transcriptase (Promega, Madison, WI, USA), 400 µM dNTP mixture, and 1× AMV RT buffer at 42°C for 45 min following a 5-min incubation at room temperature. Then, 5 µL cDNA was added to 45 µL PCR mixture to obtain a final volume of 50 µL and a concentration of 500 µM for the PCR primer, 200 µM for dNTP mixture, 1.5 mM for $MgCl_2$, 1× Taq DNA polymerase buffer, and 0.5 U for Taq DNA polymerase (Thermo Scientific, Vilnius, Lithuania). The reaction conditions consisted of an initial denaturation step at 95°C for 3 min, followed by 40 cycles of amplification (95°C for 30 sec, 48°C for 30 sec, 72°C for 2 min) and terminated at 72°C for 8 min.

We subjected 0.1 µg of cDNA to enzymatic fragmentation and adaptor ligation (NEBNext Fast DNA Fragmentation & Library Prep Set for Ion Torrent kit, New England Biolabs, Ipswich, MA, USA). The barcoded adaptors were retrieved from the Ion Xpress Barcode Adapters (Life Technologies, Carlsbad, CA, USA). The resulting cDNA libraries were measured on an Qubit 2.0 device using the Qubit dsDNA BR Assay kit (Invitrogen, Eugene, OR, USA). The emulsion PCR that produced clonally amplified libraries was carried out according to the manufacturer’s protocol using the Ion PGM Template kit on an OneTouch v2 instrument. Enrichment of the templated beads (on an Ion One Touch ES machine) and further steps of presequencing setup were performed according to the

200-bp protocol of the manufacturer. The sequencing protocol recommended for Ion PGM Sequencing Kit on an 316 chip was strictly followed (*2*,*3*).

Determination of the Termini of Genomic RNA

To obtain the true sequence of the genome segment ends, a short oligonucleotide (PC3), phosphorylated at the 5′ end and blocked at the 3′ end with dideoxy cytosine, was ligated to the 3′ ends of the genomic RNA in the nucleic acid extract (*4*,*5*). In brief, 5 µL total RNA was combined with 25 µL RNA ligation mixture (consisting of 3.5 µL nuclease free water, 2 µL of 20 µM PC3, 12.5 µL of 34% (w/v) polyethylene glycol 8000, 3 µL ATP, 3 µL 10X T4 RNA Ligase buffer and 10 U T4 RNA Ligase I (New England Biolabs, Ipswich, MA, USA) and then incubated at 17°C for 16 h. Following the incubation, the RNA was extracted by using the QIAquick Gel Extraction Kit (QIAGEN, Hilden, Germany). Binding of RNA to silica-gel column was performed in the presence of 150 µL QG buffer from the extraction kit and 180 µL isopropanol. All subsequent steps were performed according to the manufacturer's instructions.

Five microliters ligated RNA was heat-denatured in the presence of 1 µL of 20 µM primer (PC2, which is complementary to the PC3 oligonucleotide ligated to the 3′ end) at 95°C for 5 min and then placed on ice slurry. The reverse transcription mixture contained 14 µL nuclease free water, 6 µL 5× First Strand Buffer, 1 µL of 10 µM dNTP mixture, 1 µL 0.1M dTT, 20 U RiboLock RNase Inhibitor (Thermo Scientific, Vilnius, Lithuania), and 300 U SuperScript III Reverse Transcriptase (Invitrogen, Eugene, OR, USA). This mixture was added to the denatured ligated RNA and incubated at 25°C for 5 min and then 50°C for 60 min. The reaction was stopped at 70°C for 15 min (*6*).

Subsequently, 2 µL cDNA was added to the PCR mixture, which consisted of 17 µL nuclease-free water, 1 µL of 10 µM dNTP mixture, 2,5 µL 10× DreamTaq Green Buffer (including 20 mM $MgCl_2$), and 2 µL of 20 µM primer pair (i.e., 1 µL PC2 and 1 µL gene-specific primer; data not shown) and 2.5 U DreamTaq DNA polymerase (Thermo Scientific, Vilnius, Lithuania). The thermal profile consisted of the following steps: 95°C 3 min 40 cycles of 95°C 30 sec, 42°C 30 sec. 72°C 2 min final elongation at 72°C for 8

min. The PCR products were visualized on 1% agarose gel electrophoresis, and bands of the expected sizes were excised and cleaned up with Geneaid Gel/PCR DNA fragments Extraction Kit (Geneaid, Taipei, Taiwan).

Subsequently, amplicons were subjected to Sanger sequencing with the PCR primers by using the BigDye Terminator v1.1 Cycle Sequencing Kit (Applied Biosystems, Austin, TX, USA). Ethanol precipitated products were run on an ABI PRISM 310 Genetic Analyzer.

Sanger Sequencing of the VP7 Gene

Because of the significant sequence heterogeneity identified between the VP7 gene of KE135/2012 and KE528/2012, it seemed relevant to confirm the semiconductor sequencing results by using traditional sequencing. Therefore, the whole-genome segment encoding the VP7 gene was sequenced for both strains. cDNA production, amplification and Sanger sequencing were carried out with sequence specific primers (data not shown) designed based on the Ion Torrent sequence reads. The experimental protocol was essentially the same as described in the previous section describing the determination of genome segment termini.

Reference List for the Laboratory Methods Section.

1. Djikeng A, Halpin R, Kuzmickas R, Depasse J, Feldblyum J, Sengamalay N, et al. Viral genome sequencing by random priming methods. BMC Genomics. 2008;9:5. PubMed http://dx.doi.org/10.1186/1471-2164-9-5

2. Papp H, Marton S, Farkas SL, Jakab F, Martella V, Malik YS, et al. Classification and characterization of a laboratory chicken rotavirus strain carrying G7P[35] neutralization antigens on the genotype 4 backbone gene configuration. Biologicals. 10.1016/j.biologicals.2014.08.004. PubMed

3. Dóró R, Mihalov-Kovács E, Marton S, László B, Deák J, Jakab F, et al. Large-scale whole genome sequencing identifies country-wide spread of an emerging G9P[8] rotavirus strain in Hungary, 2012. Infect Genet Evol. 2014;28:495–512. PubMed http://dx.doi.org/10.1016/j.meegid.2014.09.016

4. Lambden PR, Cooke SJ, Caul EO, Clarke IN. Cloning of noncultivatable human rotavirus by single primer amplification. J Virol. 1992;66:1817–22. PubMed

5. Potgieter AC, Page NA, Liebenberg J, Wright IM, Landt O, van Dijk AA. Improved strategies for sequence-independent amplification and sequencing of viral dsRNA genomes. J Gen Virol. 2009;90:1423–32. PubMed http://dx.doi.org/10.1099/vir.0.009381-0

6. Bányai K, Dandár E, Dorsey KM, Mató T, Palya V. The genomic constellation of a novel avian orthoreovirus strain associated with runting-stunting syndrome in broilers. Virus Genes. 2011;42:82–9. PubMed http://dx.doi.org/10.1007/s11262-010-0550-z

Technical Appendix Table 2. Percentile nucleotide (nt) and amino acid (aa) sequence based identities between the novel canine rotavirus (RV) strain, KE135/2012, and reference RVA-RVD and RVF-RVH strains*

	RVA		RVB		RVC		RVD		RVF		RVG		RVH	
Gene	nt	aa	nt	aa	nt	aa	nt	aa	nt	aa	nt	aa	nt	aa
VP1	40–41	24	57–59	53–54	40–41	23	41	23	42	24	60–61	55	61–62	57–58
VP2	36	15	51–52	42	37–38	15	36	13	38	15	53	41	55–56	46
VP3	38–40	17	49–50	32	37–38	16–17	38	18	38	17	47–48	32–33	52	38–39
VP4	36–37	14–15	43	24–27	36	14–16	36	15	38	15	43–45	25–28	42	24–26
VP6	33–34	14–15	49–50	37	36–37	17–18	37	16	33	12	49–52	35–37	54	45–46
VP7†	38–39	18–20	46–48	29	35–36	16	37	17	37	15	43–45	24–26	44–45	27–28
NSP1	31–32	11	35–37	15–16	32–33	<10	34	13	34	11	38–39	18–20	48	30
NSP2	36–37	19–20	52–53	42	38	20	39	19	37	20	52–53	39–41	52	41
NSP3	38–39	18–19	42	24–26	36–37	14	39	19	34	14	43	19–22	40	20
NSP4‡	35–39	10–15	35–39	15	35–39	14–15	35	11	40	19	36–38	17	37	14–15
NSP5	33–34	10–11	45–46	24–27	32–33	10–12	30	11	35	12	46–48	27–30	47–48	28–29

*The Muscle algorithm within the Translator X (*1*) online platform was used to obtain codon-based multiple alignments. Nucleotide and deduced amino acid sequence alignments were visualized in GeneDoc (*2*), whereas sequence distances were calculated with the MEGA6 program using the P distance algorithm (*3*). Results obtained by this method were used to calculate sequence identity values.
†The VP7 gene was sequenced for both RVI strains by traditional methods as well. Of note is that Ion Torrent and Sanger sequencing results were congruent.
‡Assignment of the NSP4 was not possible by homology search. However, structure-based analysis identified putative helical transmembrane (site aa 47–64 and/or 71–88) and coiled coil region (site aa 142–168) and predicted a glycosylation site (motif, NGS; site aa 31).

References to Technical Appendix Table 2

1. Abascal F, Zardoya R, Telford MJ. TranslatorX: multiple alignment of nucleotide sequences guided by amino acid translations. Nucleic Acids Res. 2010;38:W7–13. PubMed http://dx.doi.org/10.1093/nar/gkq291

2. Nicholas KB, Nicholas HB Jr, Deerfield DW II. GeneDoc: analysis and visualization of genetic variation. Embnet News. 1997;4:14.

3. Tamura K, Stecher G, Peterson D, Filipski A, Kumar S. MEGA6: Molecular Evolutionary Genetics Analysis version 6.0. Mol Biol Evol. 2013;30:2725–9. PubMed http://dx.doi.org/10.1093/molbev/mst197

The 5′ end sequence of the VP2 gene of KE528/2012

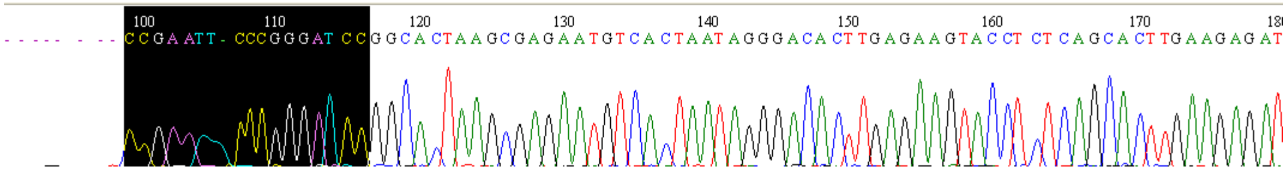

The 3′ end sequence of the VP2 gene of KE528/2012

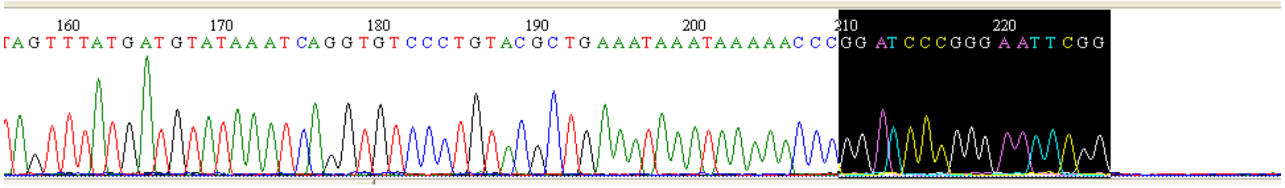

Technical Appendix Figure 1. 5′ and 3′ termini confirmed by Sanger sequencing. To illustrate the sequencing results, an example is inserted below. The ligated oligonucleotide sequence at the 3′ ends of the genomic RNA is shown with dark background. Please note that numbers above the peaks indicate the base position in the chromatogram and not the base position in the genome segment.

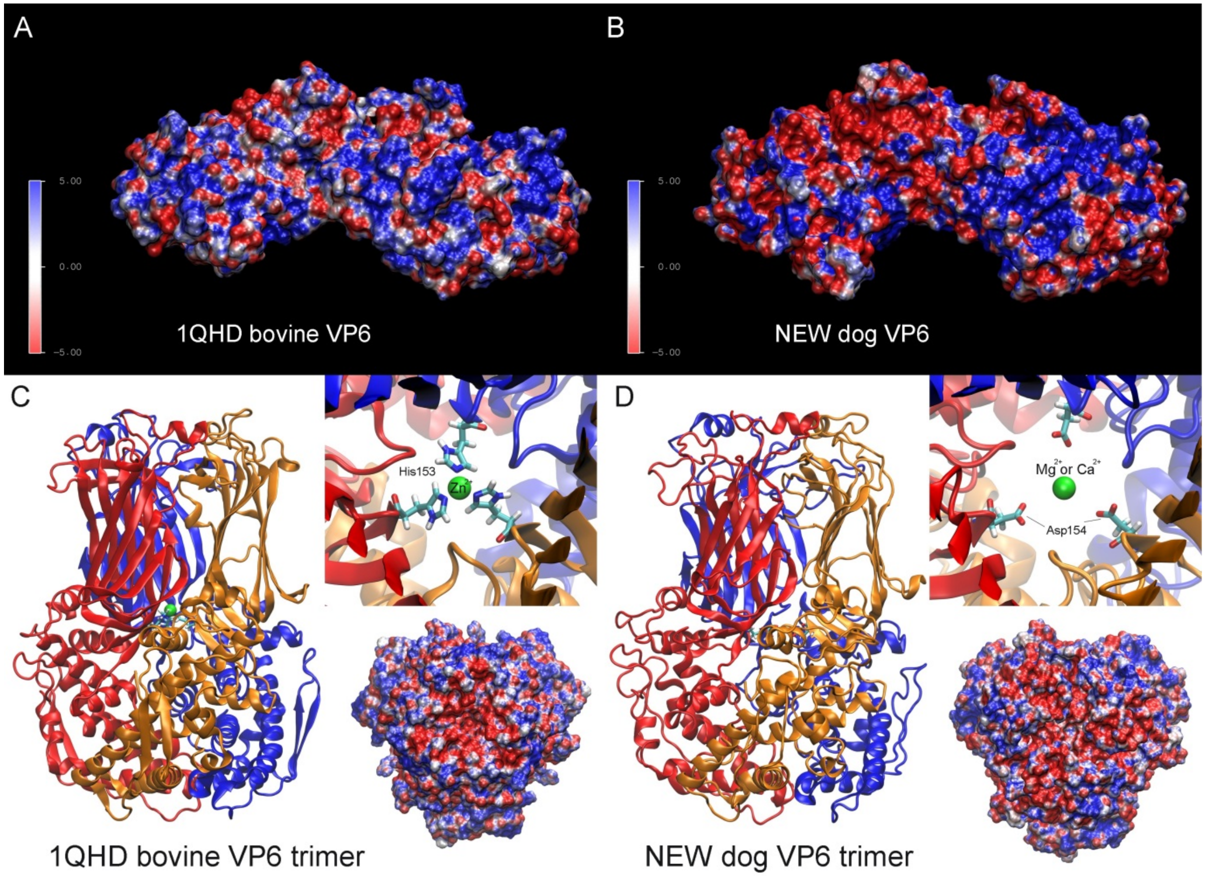

Technical Appendix Figure 2. Additional insight into the structure of the VP6 protein and its homotrimer form. The protein structure was generated with I-TASSER (*1*) by using the following experimentally determined templates (referring PDB ID codes): 1QHD (VP6, bovine RVA strain RF), 3KZ4 (VP6, bovine RVA strain UK), 3SMT (Human SET domain-containing protein3), 1B5Q (Polyamine oxidase from *Zea mays*), 1XPQ (Polyamine oxidase from yeast) and 1SEZ (Protoporphyrinogen IX oxidase from tobacco). A VP6 trimer was created from the generated VP6 model using the biologic assembly coordinate of the main template, the bovine RVA VP6 protein trimer (PDB ID: 1QHD). The model structures were refined with the Schrödinger molecular modeling software package (*2*) to eliminate the steric conflicts between the protein side chain atoms. Pairwise protein sequence alignment was calculated with the NeedleP tool of the SRS bioinformatics software package. Electrostatic

potential maps were calculated with Adaptive Poisson–Boltzmann Solver (APBS) version 1.3 by using the linearized Poisson–Boltzmann method with a dielectric constant of 78 and 2 for the water solvent and protein core, respectively. The partial charges for the electrostatic potential calculations were calculated with PDB2PQR (*3–5*). Molecular graphics and sequence alignment visualization were prepared by using VMD version 1.9.1 and the Multiple Sequence Viewer of the Schrödinger Suite, respectively (*6*). Electrostatic view of the bovine VP6 (A) and the new canine VP6 (B) rotavirus coat protein surfaces. Colors: red, regions with potential value less than –5.0 kT; white, 0.0; blue, greater than +5.0 kT. Comparison between bovine (C) and the canine (D) VP6 trimers. The central metal ion binding sites are indicated on the right top insets of C and D. The outer antigenic surface of the VP6 trimers (right lower insets) are colored by electrostatic potential distribution. Previous studies demonstrated that the RVA VP6 trimer is stabilized by Zn^{2+} located at the center of the complex on the 3-fold axis (C). The bound Zn^{2+} is coordinated by His153 from each of the 3 VP6 subunits. Interestingly, the novel canine rotavirus VP6 protein possessed no His around this location. Instead, a negatively charged amino acid residue, Asp, was found in position 154. Negatively charged amino acids (such as Asp and Glu) usually take part in Mg^{2+}, Mn^{2+} and Ca^{2+} ion coordination but not Zn^{2+} ion. Based on this finding we assume that a metal ion other than Zn^{2+} (e.g., Mg^{2+} or Ca^{2+}) may be coordinated by Asp154 in the center of the canine RVI VP6 capsomere to stabilize the trimer form (Panel D). The question whether if this finding might have implications for virion stability or resource use within the infected cell during virion assembly is open.

Reference List for Technical Appendix Figure 2

1. Zhang Y. I-TASSER server for protein 3D structure prediction. BMC Bioinformatics. 2008;9:40. PubMed http://dx.doi.org/10.1186/1471-2105-9-40

2. Suite S. New York: Schrödinger, LLC; 2013.

3. Baker NA, Sept D, Joseph S, Holst MJ, McCammon JA. Electrostatics of nanosystems: application to microtubules and the ribosome. Proc Natl Acad Sci U S A. 2001;98:10037–41. PubMed http://dx.doi.org/10.1073/pnas.181342398

4. Gilson MK, Sharp KA, Honig B. Calculating electrostatic interactions in biomolecules: method and error assessment. J Comput Chem. 1987;9:327–35. http://dx.doi.org/10.1002/jcc.540090407

5. Dolinsky TJ, Nielsen JE, McCammon JA, Baker NA. PDB2PQR: an automated pipeline for the setup, execution, and analysis of Poisson-Boltzmann electrostatics calculations. Nucleic Acids Res. 2004;32(Web Server issue):W665–7.

6. Humphrey W, Dalke A, Schulten K. VMD - Visual Molecular Dynamics. J Mol Graph. 1996;14:33–8. PubMed http://dx.doi.org/10.1016/0263-7855(96)00018-5

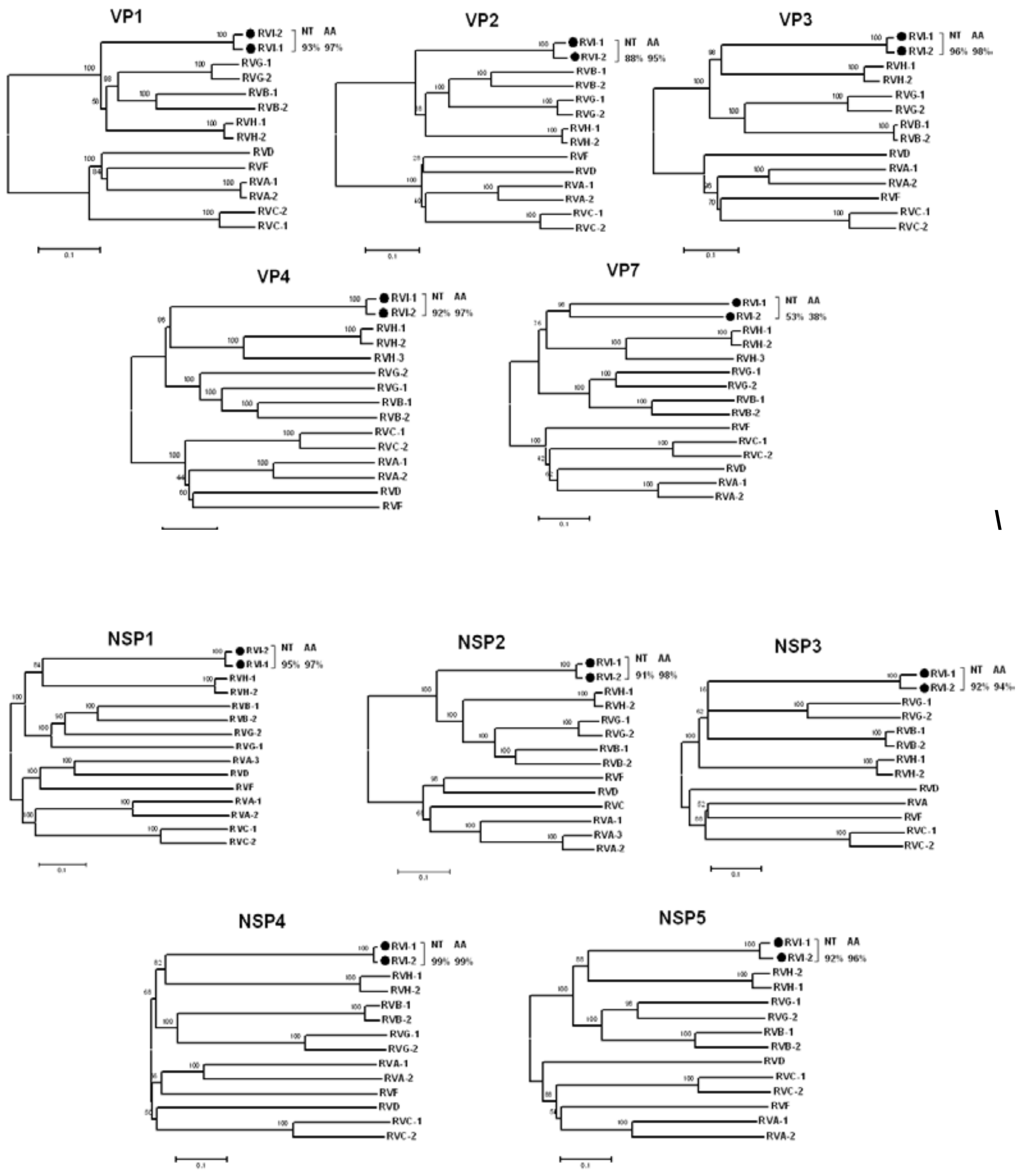
VP1
RVI-2
RVI-1
NT AA
93% 97%
RVG-1
RVG-2
RVB-1
RVB-2
RVH-1
RVH-2
RVD
RVF
RVA-1
RVA-2
RVC-2
RVC-1
VP2
RVI-1
RVI-2
NT AA
88% 95%
RVB-1
RVB-2
RVG-1
RVG-2
RVH-1
RVH-2
RVF
RVD
RVA-1
RVA-2
RVC-1
RVC-2
VP3
RVI-1
RVI-2
NT AA
96% 98%
RVH-1
RVH-2
RVG-1
RVG-2
RVB-1
RVB-2
RVD
RVA-1
RVA-2
RVF
RVC-1
RVC-2
VP4
RVI-1
RVI-2
NT AA
92% 97%
RVH-1
RVH-2
RVH-3
RVG-2
RVG-1
RVB-1
RVB-2
RVC-1
RVC-2
RVA-1
RVA-2
RVD
RVF
VP7
RVI-1
RVI-2
NT AA
53% 38%
RVH-1
RVH-2
RVH-3
RVG-1
RVG-2
RVB-1
RVB-2
RVF
RVC-1
RVC-2
RVD
RVA-1
RVA-2
NSP1
RVI-2
RVI-1
NT AA
95% 97%
RVH-1
RVH-2
RVB-1
RVB-2
RVG-2
RVG-1
RVA-3
RVD
RVF
RVA-1
RVA-2
RVC-1
RVC-2
NSP2
RVI-1
RVI-2
NT AA
91% 98%
RVH-1
RVH-2
RVG-1
RVG-2
RVB-1
RVB-2
RVF
RVD
RVC
RVA-1
RVA-3
RVA-2
NSP3
RVI-1
RVI-2
NT AA
92% 94%
RVG-1
RVG-2
RVB-1
RVB-2
RVH-1
RVH-2
RVD
RVA
RVF
RVC-1
RVC-2
NSP4
RVI-1
RVI-2
NT AA
99% 99%
RVH-1
RVH-2
RVB-1
RVB-2
RVG-1
RVG-2
RVA-1
RVA-2
RVF
RVD
RVC-1
RVC-2
NSP5
RVI-1
RVI-2
NT AA
92% 96%
RVH-2
RVH-1
RVG-1
RVG-2
RVB-1
RVB-2
RVD
RVC-1
RVC-2
RVF
RVA-1
RVA-2

Technical Appendix Figure 3. Phylogenetic trees obtained for the VP1 to VP4, VP7, NSP1 to NSP5 proteins with representative strains of RVA to RVH. RVI-1, and RVI-2 represents KE135/2012 and KE528/2012, respectively. Alignments were created by using the BLOSUM62 algorithm as implemented at the Multalin website (http://multalin.toulouse.inra.fr/multalin/). Phylogenetic trees were prepared by using the neighbor-joining method. Bootstrap values are shown at the branch nodes. Nucleotide and amino acid identities between KE135/2012 and KE528/2014 are show on the right. Of note is the low sequence homology within the VP7 of RVI strains, KE135/2012 and KE528/2014. Such limited VP7 sequence identity values classify RVA-RVC rotaviruses into different G genotypes. Therefore, we tentatively assigned the 2 RVI strains into 2 different G types, G1 and G2 (see the main text). In the other genes, the RVI strains in our study most likely share the respective genotype specificity.

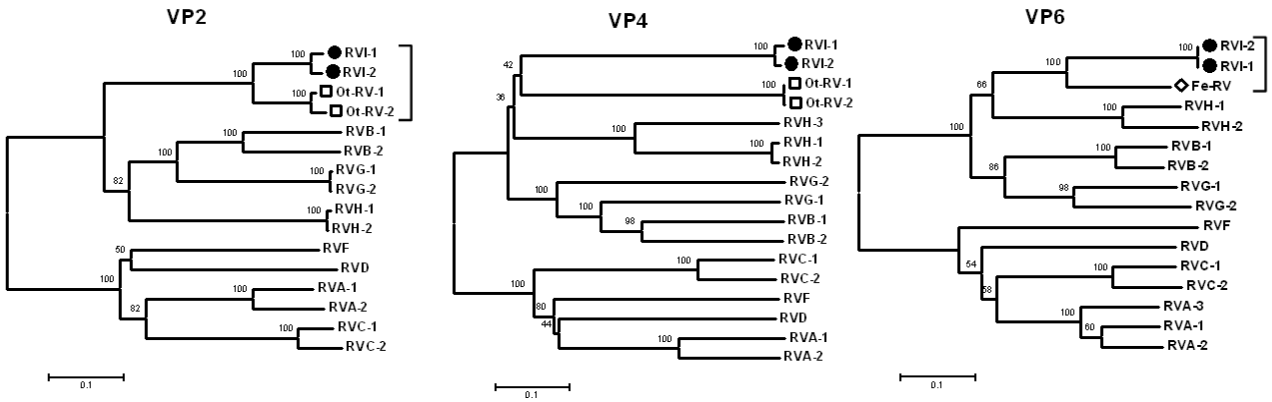

Technical Appendix Figure 4. Phylogenetic trees obtained for the partial sequences using unusual feline and otarine RV gene sequences. The alignments of the VP2, VP4, and VP6 proteins encompassed ≈160, ≈310, and ≈70 aa long sequences.
